# B-mode ultrasound assessment of pupillary function: Feasibility, reliability and normal values

**DOI:** 10.1371/journal.pone.0189016

**Published:** 2017-12-06

**Authors:** Felix A. Schmidt, Klemens Ruprecht, Florian Connolly, Matthew B. Maas, Friedemann Paul, Jan Hoffmann, Lutz Harms, Stephan J. Schreiber

**Affiliations:** 1 Department of Neurology, Charité – Universitätsmedizin Berlin, Berlin, Germany; 2 NeuroCure Clinical Research Center, Charité – Universitätsmedizin Berlin, Berlin, Germany; 3 Department of Neurology, Feinberg School of Medicine, Northwestern University, Chicago, Illinois, United States of America; 4 Experimental and Clinical Research Center, Max Delbrueck Center for Molecular Medicine and Charité – Universitätsmedizin Berlin, Berlin, Germany; 5 Department of Systems Neuroscience, University Medical Center Hamburg-Eppendorf, Hamburg, Germany; 6 Department of Neurology, Asklepios Fachklinikum Brandenburg, Brandenburg an der Havel, Germany; Tokai University, JAPAN

## Abstract

**Purpose:**

To evaluate B-mode ultrasound as a novel method for the examination of pupillary function and to provide normal values for the pupillary reflex as assessed by B-mode ultrasound.

**Methods:**

100 subjects (49 female, 51 male, mean [range] age 51 [18–80 years]) with no history of ophthalmologic disease, no clinically detectable pupillary defects, and corrected visual acuity ≥ 0.8 were included in this prospective observational study. B-mode ultrasound was performed with the subjects eyes closed using an Esaote-Mylab25 system according to current guidelines for orbital insonation. A standardized light stimulus was applied.

**Results:**

The mean ± standard deviation left and right pupillary diameters (PD) at rest were 4.7 ± 0.8 and 4.5 ± 0.8 mm. Following an ipsilateral light stimulus (L_stim)_, left and right constricted PD were 2.8 ± 0.6 and 2.7 ± 0.6 mm. Following a contralateral L_stim_, left and right constricted PD were 2.7 ± 0.6 and 2.6 ± 0.5 mm. Left and right pupillary constriction time (PCT) following ipsilateral L_stim_ were 970 ± 261.6 and 967 ± 220 ms. Left and right PCT following a contralateral L_stim_ were 993.8 ± 192.6 and 963 ± 189.4 ms. Patient age was inversely correlated with PD at rest and with PD after ipsilateral and contralateral L_stim_ (all p<0.001), but not with PCT.

**Conclusions:**

B-mode ultrasound is a simple, rapid and objective method for the quantitative assessment of pupillary function, which may prove useful in a variety of settings where eyelid retraction is impeded or an infrared pupillometry device is unavailable.

## Introduction

Assessment of pupillary shape and size as well as of the pupillary light reflex (PLR) is a standard diagnostic procedure in ophthalmological and neurological examinations [1]. Clinical examination of pupillary function typically includes estimation of pupillary diameters (PD) and testing the PLR with a penlight [2]. Accurate clinical assessment of pupillary function may be impeded by inability to retract the eyelid due to swelling or lack of cooperation, environmental light exposure and examiner expertise. Moreover, subjective estimates of pupil sizes are not sufficiently reliable to allow for longitudinal inter-rater comparisons. Alternative methods for the objective assessment of pupillary function include sophisticated diagnostic systems like video assessment or infrared pupillometry devices [3]. However, these instruments are rarely available outside of specialized centers.

In contrast, B-mode ultrasound is a simple and widely available noninvasive imaging technique. To the best of our knowledge, ocular ultrasound for examination of pupillary function has thus far been reported in a single patient with an ocular trauma [4]. The objectives of this study were to establish the feasibility of measuring PD and pupillary constriction times (PCT) with B-mode ultrasound, and to provide normal values for the PLR in a representative set of patients with no ophthalmologic disease.

## Materials and methods

### Study design

In this prospective observational study 100 participants of 4 different age groups were recruited from hospital staff and from the routine vascular patient population of the neurosonological lab at the Department of Neurology, Charité Campus Mitte, Charité –Universitätsmedizin Berlin. 25 subjects were enrolled in each of 4 pre-specified age groups. The main outcome parameters assessed with B-mode ultrasound were PD, PCT and pupillary constriction amplitude.

### Study participants

Inclusion criteria were age ≥ 18 and ≤ 80 years and a bilateral best corrected visual acuity (VA) ≥ 0.8 (20/25 in United States customary units). Furthermore, clinical examination of pupillary function had to be normal. Exclusion criteria were any clinically detectable pupillary dysfunction, visual field defects or oculomotor abnormalities. Patients with a history of any ocular disease (e.g. glaucoma, cataract, macular degeneration or diabetic retinopathy), neurological diseases that can potentially cause optic neuropathy or optic atrophy (e.g. multiple sclerosis, clinically isolated syndrome, neuromyelitis optica spectrum disorder, chronic relapsing inflammatory optic neuritis or Leber hereditary optic neuropathy), or any autoimmune or systemic diseases that might have an effect on the autonomic nervous system (e.g. Guillain-Barré syndrome, viral and limbic encephalitis, multiple system atrophy, sarcoidosis and systemic lupus erythematosus), patients that had undergone any type of ocular surgery or laser treatment in the past and patients taking topical or systemic medications potentially affecting pupillary function were likewise excluded from the study.

### Visual acuity testing

Best corrected visual acuity was determined under standardized light conditions using a Snellen Chart [5]. Subjects read Sloan letters of different sizes from a distance of 3 m separately with each eye.

### B-mode ultrasound technique

**Fig 1:**
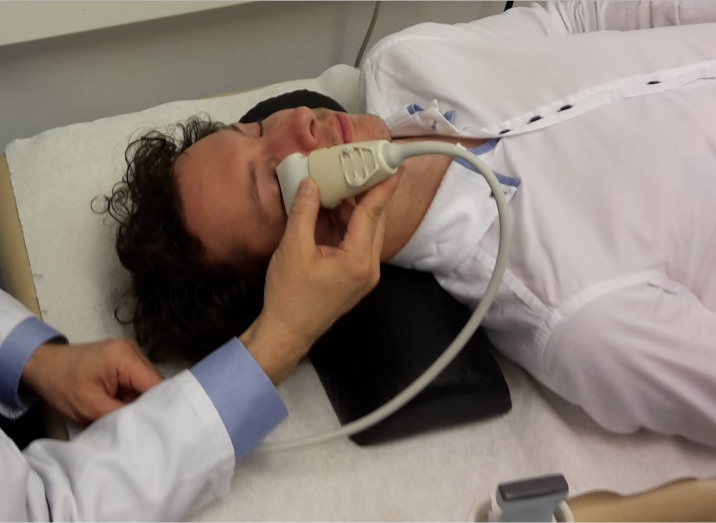
Assessment of pupillary function by B-mode ultrasound. Position of the proband and ultrasound probe.

**Fig 2:**
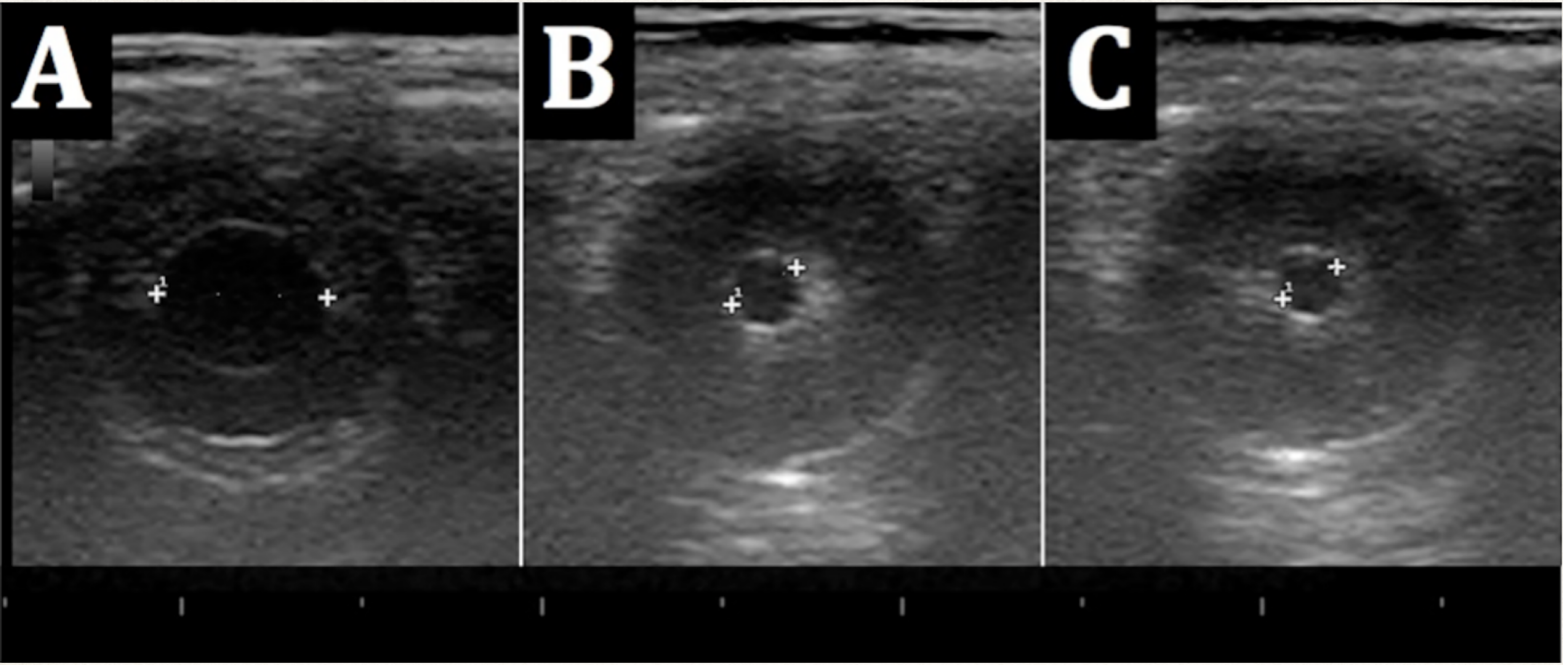
Example of pupillary diameter [PD] assessment in the closed eye by B-mode ultrasound. (A) PD at rest under standard dimmed light conditions of the ultrasound study room. (B) PD during ipsilateral light stimulus [L_stim_]. (C) PD during contralateral L_stim_. Crosses represent the markers set by the examiner for measuring the PD.

All subjects were studied in supine position under standardized dimmed light conditions (room lighting 30 Lux) of the ultrasound examination room with the examiner sitting at the head side (Fig 1). To adapt to the light level, study participants spent at least 10 minutes in the room before testing. All insonations were performed by the same investigator (SJS) with the subject’s eyes closed using an Esaote Mylab 25 system equipped with a 10 MHz linear array probe. Power settings were reduced to minimum, according to the ALARA (as low as reasonably achievable) insonation approach, and we adhered to current guidelines for orbital insonation [6]. B-mode settings were adjusted for near-field eye examination. Each pupil was visualized with the probe positioned flatly on the lower eyelid, leveraging Bell’s phenomenon. For assessment of the PLR, subjects had the eyes closed, a penlight was activated by an assisting investigator approximately 2 cm in front of each closed eye and the light reaction of each pupil was digitally documented (Fig 2). In every examination the same standard penlight was used with a luminous emittance of 70,000 Lux and a stimulus time of 2 seconds to ensure constant wavelength, intensity and duration of the light stimulus as the PLR is dependent on these properties of light. Each assessment was performed in exactly the same order starting by measuring the PD of the left eye at rest as well as during ipsilateral and contralateral light stimulus (L_stim_) and subsequently performing the same examinations on the right eye. The extent of pupillary constriction was then calculated as the difference between PD at rest and during ipsilateral and contralateral L_stim_.

### Data analysis

PD were manually assessed in a frozen still image of the pupil which was then digitally stored. Using the build-in measuring tool of the ultrasound system, the largest PD at rest and the smallest PD after L_stim_ were labeled. Analysis of the PCT, defined as the time interval (measured in milliseconds) between the maximum and the minimum PD during L_stim_, was performed by recording 5 second video sequences of a second ipsi- and contralateral L_stim_, approximately 2 min after the PD analysis. The videos were transferred to a computer and the PCT was manually determined using the software AVSVideoConverter9.2 (Online Media Technologies Ltd. London, UK).

### Statistical analysis

Statistical analyses were performed using SPSS 23.0 (IBM SPSS Statistics, New York, USA). Figures were created with GraphPadPrism 6.0 (GraphPad Software Inc, California, USA). PD and PCT are reported as mean ± standard deviation. The results of the PLR assessment were correlated to age using Pearson correlation. One-way analysis of variance (ANOVA) was used to compare means of PD results between different age groups. Bland-Altman-analysis was performed to assess test-retest reliability.

The study was approved by the institutional review board of Charité - Universitätsmedizin Berlin (EA1/190/15) and was conducted in accordance to the Declaration of Helsinki in its currently applicable version and applicable German laws. All participants provided written informed consent. The individual in this manuscript displayed on Fig 1 has given written informed consent (as outlined in PLOS consent form) to publish these case details.

## Results

### Study participants

A total of 103 subjects were recruited into the study. Three (2.9%) individuals did not tolerate ultrasound examination due to an unpleasant feeling of the ultrasound probe touching the closed eye lid and could therefore not be evaluated. The 100 individuals (49 female, 51 male) available for the final analysis were divided into 4 age groups, each group consisting of 25 individuals. Group 1 (G1) ranged from 18–35 years, group 2 (G2) from 36–50 years, group 3 (G3) from 51–65 years and group 4 (G4) from 66–80 years (Table 1).

**Table 1:** Patient demographics and pupillary measurement results.

	All (n = 100)	G1 Age 18–35 (n = 25)	G2 Age 36–50 (n = 25)	G3 Age 51–65 (n = 25)	G4 Age 66–80 (n = 25)
**Demographics**					
**Age, years**	50 ± 17	27 ± 4	44 ± 5	59 ± 5	72 ± 4
**Gender (f/m)**	49/51	16/9	15/10	13/12	6/19
**PD at rest**					
**Left eye (mm)**	4.7 ± 0.8	5.2 ± 0.7	4.9 ± 0.7	4.5 ± 0.5	4.1 ± 0.8
**Right eye (mm)**	4.5 ± 0.8	5.1 ± 0.6	4.8 ± 0.8	4.4 ± 0.6	3.8 ± 0.7
**PD during ipsilateral light stimulus**					
**Left eye (mm)**	2.8 ± 0.6	3.2 ± 0.6	2.9 ± 0.5	2.7 ± 0.4	2.5 ± 0.6
**Right eye (mm)**	2.7 ± 0.5	3.1 ± 0.4	2.7 ± 0.5	2.6 ± 0.5	2.3 ± 0.4
**Difference between PD at rest and during ipsilateral light stimulus**					
**Left eye (mm)**	1.8 ± 0.4	2.0 ± 0.3	1.9 ± 0.4	1.8 ± 0.4	1.6 ± 0.4
**Right eye (mm)**	1.8 ± 0.4	1.9 ± 0.4	2.0 ± 0.4	1.8 ± 0.3	1.5 ± 0.4
**PD during contralateral light stimulus**					
**Left eye (mm)**	2.7 ± 0.6	3.1 ± 0.6	2.8 ± 0.5	2.6 ± 0.5	2.3 ± 0.5
**Right eye (mm)**	2.6 ± 0.5	2.9 ± 0.5	2.6 ± 0.4	2.5 ± 0.5	2.2 ± 0.4
**Difference between PD at rest and during contralateral light stimulus**					
**Left eye (mm)**	2.0 ± 0.4	2.1 ± 0.4	2.1 ± 0.4	1.9 ± 0.4	1.8 ± 0.5
**Right eye (mm)**	2.0 ± 0.5	2.1 ± 0.5	2.2 ± 0.5	1.9 ± 0.3	1.6 ± 0.5
**Pupillary constriction time after ipsilateral light stimulus**					
**Left eye (ms)**	970 ± 262	1000 ± 290	1000 ± 207	960 ± 250	920 ± 299
**Right eye (ms)**	967 ± 220	1004 ± 210	940 ± 191	912 ± 183	1016 ± 237
**Pupillary constriction time after contralateral light stimulus**					
**Left eye (ms)**	994 ± 193	992 ± 138	1036 ± 193	932 ± 206	1012 ± 233
**Right eye (ms)**	963 ± 189	984 ± 177	992 ± 178	916 ± 217	960 ± 184

Except for gender distribution all values given as mean ± standard deviation, f = female, G = group, m = male, mm = millimeter, ms = milliseconds, n = number of individuals, PD = Pupillary diameter, y = years

### B-mode ultrasound for assessment of pupillary function

As shown in Fig 2, B-mode ultrasound enabled unambiguous detection of the pupils and PD could easily be determined by manual measurement. Following examination of the pupil with eyes closed at rest (Fig 2A), an ipsilateral (Fig 2B) or contralateral (Fig 2C) L_stim_ in front of the closed eyes resulted in prompt pupillary constriction. The total examination time per study participant was approximately 5 minutes.

### Pupillary diameters at rest and during light stimulus

Table 1 summarizes the findings for PDs at rest and after ipsilateral and contralateral L_stim_ for the entire study population as well as for the four defined age groups. None of the 100 study subjects had a PD difference between both eyes at rest of ≥ 1 mm and was thus considered to have physiological anisocoria. Constriction in each eye following ipsilateral and contralateral stimulation was similar (Pearson r = 0.83 for the left eye and r = 0.90 for the right eye; both p<0.001). The extent of constriction to L_stim_ when comparing the left and the right eye was also correlated for both ipsilateral and contralateral stimuli (Pearson r = 0.54 for the ipsilateral L_stim_ and r = 0.56 for the contralateral L_stim_; both p<0.001).

### Pupillary constriction times

The PCTs after ipsilateral and contralateral light stimulus were similar between both eyes and after ipsilateral and contralateral light stimulation (Table 1).

### Correlation of pupillary parameters with age

**Fig 3:**
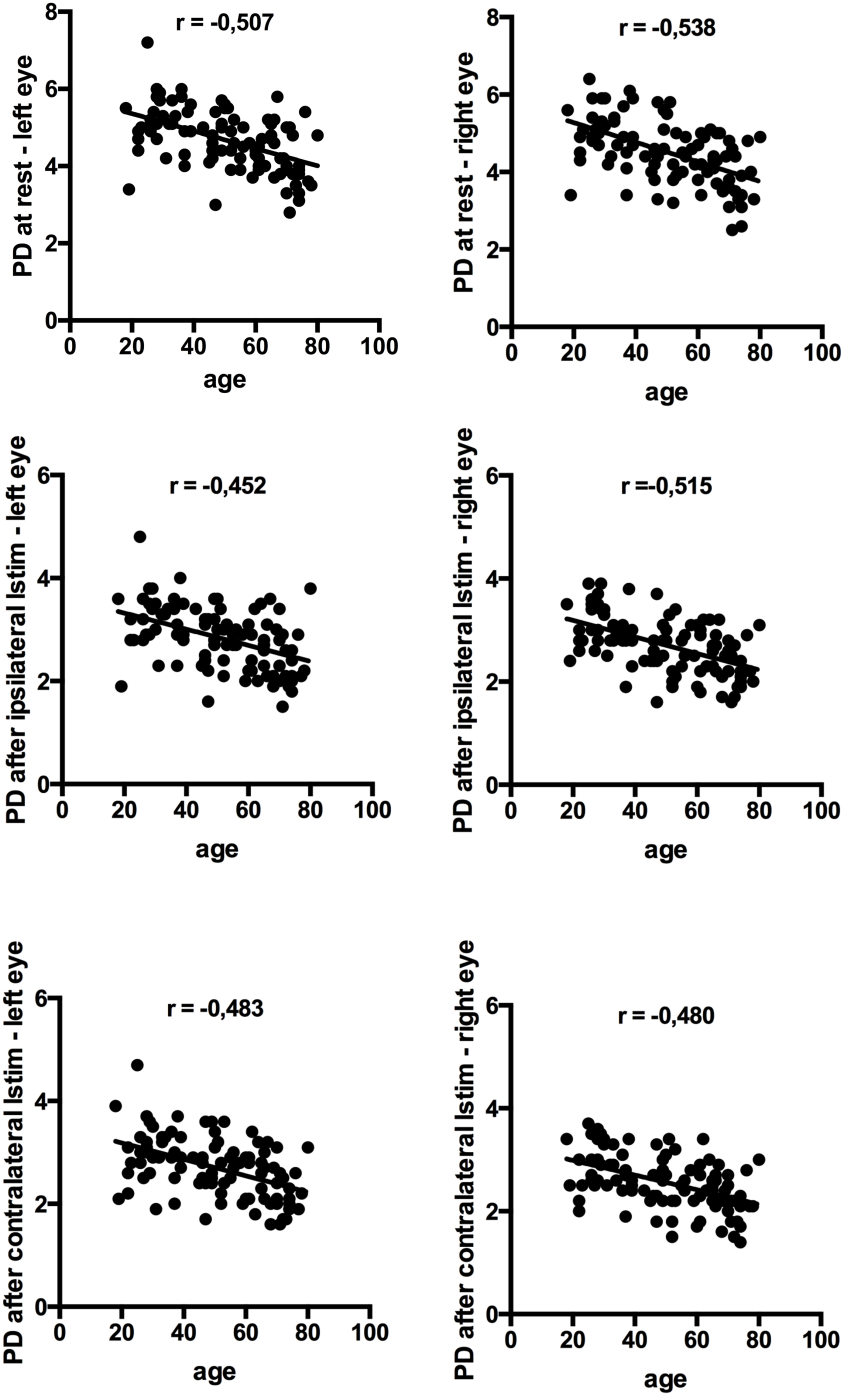
Correlations of pupillary diameter (PD) with age. Y-axis: all PD’s in millimeters, x-axis: age in years. L_stim_ = light stimulus, r = Pearson correlation coefficient, p < 0.001.

PDs at rest and during ipsi- and contralateral L_stim_ significantly decreased with age, with Pearson correlation coefficients ranging between r = -0,45 and r = -0,54 (p < 0.001, Fig 3).

By analysis of variance (ANOVA), a continuous cross-sectional decrease of PD for every 10 years cross-section increase of age could be detected. The right resting PD was on average for every additional 10 years of age 0.25mm lower (95% confidence interval 0.17–0.33mm) and the left resting PD was 0.23mm lower (95% confidence interval 0.15–0.3). The right eye ipsilateral L_stim_ PD was 0.16mm lower for every additional 10 years of age (95% confidence interval 0.11–0.21), the left eye ipsilateral L_stim_ PD was 0.16mm lower (95% confidence interval 0.09–0.22). The right eye contralateral L_stim_ PD was 0.14mm lower (95% confidence interval 0.09–0.19), the left eye contralateral L_stim_ PD was on average 0.16mm lower for every additional 10 years of age (95% confidence interval 0.1–0.22).

The extent of pupillary constriction following ipsi- and contralateral L_stim_ likewise decreased significantly with increasing age (p<0.001). No significant correlation was found between age and PCT.

### Test reproducibility

**Fig 4:**
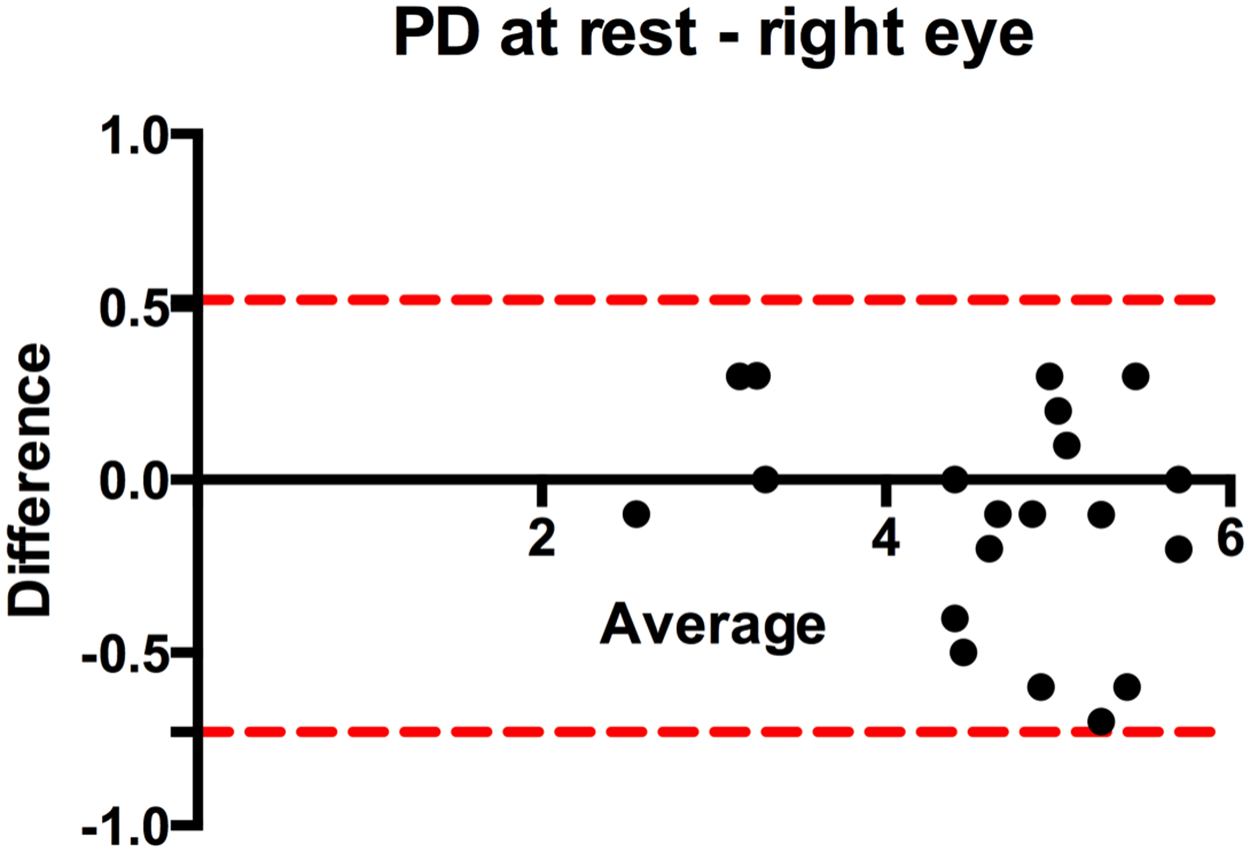
PD at rest—exemplarily for the right eye. X-axis: average of means for measurement 1 and 2 for every patient, PD in mm. Y-Axis: Differences between both measurements in mm. Upper and lower 95% limits of agreement are indicated.

For reliability analysis, measurements from the initial still images were compared with the data from the video sequences, obtained in each subject for the PCT measurements 2 minutes later. For this, every 5^th^ data set was analyzed off-line after completion of study data acquisition with the rater blinded to the initially obtained PD values. The mean (± standard deviation) difference between both measurements (bias) for the right eye was -0.11 (±0.32) mm and for the left eye -0.19 (±0.26) mm, demonstrating a good agreement between both measurements (Fig 4).

## Discussion

We here report the first systematic evaluation of B-mode ultrasound for assessment of pupillary function and provide normal values for ultrasound derived PD and PCT values for 100 subjects in different age groups. PLR assessment with ultrasound in our study was well tolerated with a dropout rate of < 3% and was rapidly acquired in five minutes during a routine clinical visit. Furthermore, PLR assessment by ultrasound had a good test-retest reliability.

Infrared video pupillometry (IVP) is an established method for assessment of the PLR that is performed by recording the pupil with an infrared video camera [7]. IVP showed superiority compared to other manually performed diagnostic tests for PLR assessment like the swinging flashlight test [8,9]. Our present data obtained by pupillary ultrasound are consistent with pupillometry data for PLR specific parameters assessed by IVP published in the literature. Prior studies with IVP have reported nearly identical values for PD (mean 5.5 vs. 4.9 mm) and constriction amplitude (mean 1.6 vs. 1.9 mm) compared to our matching age group [10–12]. Similarly, IVP studies have also reported decreasing PD with age, as found in our cohort [13]. However, a comparison of different data sets of the PLR assessed with IVP and ultrasound imposes certain difficulties as the PLR depends on light properties such as irradiance, wavelength and duration. Studies that directly compare IVP and B-mode ultrasound for PLR assessment carried out with the same light stimulus and light conditions are currently lacking and should be performed in the future to enable a direct comparison of both methods.

There are clear advantages of ocular ultrasound approach for PLR assessment compared to IVP. Ultrasound machines with 10MHz linear arrays are more widely available in hospitals and emergency departments than IVP devices, likely due to acquisition costs and a broader range of applications for ultrasound [14]. PLR assessment can be performed with the subject’s eyes closed, so examinations may still be feasible in cases in which eyelid retraction is impeded by poor cooperation or severe periorbital edema [4]. Nevertheless, the ultrasound approach currently also poses some limitations compared to IVP. IVP allows for a more detailed analysis of the PLR beyond PD and PCT, assessing pupil latency time, velocity, dilation, acceleration and photoreceptor function as well [10]. As the ultrasound examination in our study was performed with the eyes closed, it is difficult to determine the irradiance that reaches the retina and consequently to differentiate between rod, cone and melanopsin function [15]. Furthermore IVP may be less influenced by environmental lighting and requires minimal training, whereas B-mode ultrasound requires certain experience and standardized conditions. However in our experience B-mode ultrasound was a suitable tool to assess the most common PLR parameters in clinical routine.

Spontaneous pupil oscillation is a phenomenon described in literature [2,16] that was observed in several subjects in our study. A single PD measurement obtained by a frozen ultrasound image could lead to false results as pupil size may vary. Nevertheless, in our test reproducibility analysis we just detected small differences in PD’s (<0.2mm) comparing measurement at different time points, thus we conclude that spontaneous pupil oscillation does not severely bias the PLR assessment with ultrasound.

Altogether, we conclude that the assessment of the PLR with B-mode ultrasound is an innovative, widely available, time and cost effective method to document routine clinical pupillary testing. B-mode ultrasound of the eye allows for direct measurement of the PLR and its dynamic component, the PCT. Further study is needed to confirm the utility of ultrasound in detecting pathologic abnormalities in PLR. Development of automated imaging functions, similar to those incorporated into IVP devices, may reduce the technical expertise barrier as well.
